# Dalbavancin to Complete a Prolonged Antibiotic Course for Persistent *Bacillus cereus* Bacteremia: A Case Report and Literature Review

**DOI:** 10.1155/crdi/2025963

**Published:** 2025-10-03

**Authors:** Jahnavi Yetukuri, Joseph Choi, Andrew Darkow

**Affiliations:** ^1^Ernest Mario School of Pharmacy, Rutgers University, Piscataway, New Jersey, USA; ^2^Department of Pharmacy, Duke Regional Hospital, Durham, North Carolina, USA; ^3^College of Pharmacy & Health Sciences, Campbell University, Buies Creek, North Carolina, USA

## Abstract

*Bacillus cereus* is a Gram-positive, rod-shaped facultative anaerobe commonly associated with gastrointestinal infections. Although typically regarded as a blood culture contaminant, *B. cereus* can cause severe infections, including bacteremia, in high-risk patients. We report a case of a 39-year-old male with a history of intravenous drug use (IVDU) presenting with fevers and found to have *B. cereus* bacteremia. Cultures remained persistently positive for *B. cereus* despite 7 days of therapeutic vancomycin. Gentamicin was initiated for Gram-positive synergistic effect with vancomycin. This combination of gentamicin and vancomycin resulted in blood culture clearance. On discharge, he received one dose of dalbavancin and oral levofloxacin for 21 days to complete a total 4-week antibiotic course from date of blood culture clearance. This case demonstrates that dalbavancin may be a promising alternative to standard treatments for prolonged antibiotic courses of *B. cereus* infections, particularly when intravenous access is a challenge.

## 1. Introduction

*Bacillus cereus* is a Gram-positive, rod-shaped, aerobic or facultatively anaerobic, motile, spore-forming bacterium that is widely distributed throughout environmental settings [1]. While it is often characterized as a culture contaminant or cause of food poisoning, *B. cereus* has been increasingly reported to be the cause of serious and potentially fatal infections [1, 2]. Its pathogenicity is closely associated with its secretion of a variety of enzymes including hemolysins, phospholipases, emesis-inducing toxin, and pore-forming enterotoxins [1]. It also produces β-lactamase and is generally resistant to β-lactam antibiotics including cephalosporins [3]. Those most commonly infected by *B. cereus* are immunosuppressed individuals, neonates, people who inject intravenous drugs (PWID), those sustaining traumatic or surgical wounds, and patients with indwelling catheters [1]. The spectrum of infections may include bacteremia, endocarditis, meningitis, endophthalmitis, pneumonia, cutaneous infections, and food poisoning [1, 3]. Some patients may develop persistent disseminated infections or infectious complications warranting extended durations of therapy [3–5]. Early appropriate treatment of *B. cereus* is essential, with most isolates commonly being susceptible to vancomycin [6]. Aminoglycosides, carbapenems, and fluoroquinolones have also been reported in the literature to be effective [3, 6–8].

In this case report, we describe a patient who presented to the hospital with *B. cereus* bacteremia in the setting of intravenous drug use (IVDU). The patient was initially treated with vancomycin, but blood cultures remained positive following 11 days of therapy. Extensive workup for metastatic sites of infection was negative. Gentamicin was added to vancomycin on Day 11, with subsequent blood culture clearance on hospital Day 13. Vancomycin and gentamicin were continued through hospital Day 19. The discharge antibiotic selection was limited by the patient's history of IVDU. On Day 20, the patient was given a single 1500-mg dose of dalbavancin and initiated on oral levofloxacin 750 mg daily for 21 days to complete 4 weeks of therapy from first blood culture clearance. This case report highlights the potential role for dalbavancin to complete prolonged courses of parenteral antibiotic therapy for persistent *B. cereus* infections.

## 2. Case

**Figure 1:**
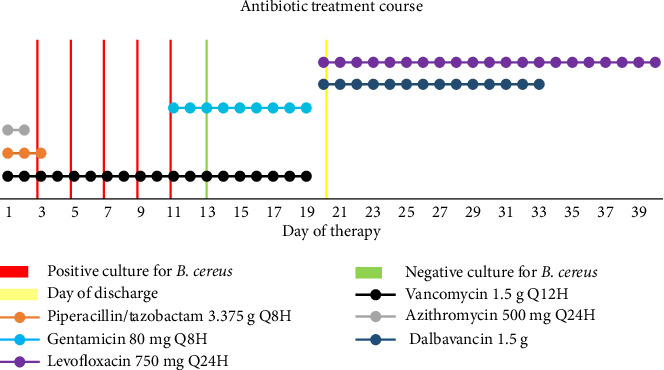
Patient's antibiotic treatment course and blood culture results.

A 39-year-old male was brought to the emergency department for evaluation of intoxication and hyperthermia. On presentation, he had a temperature of 102.2°F, heart rate of 126 beats per min, and respiratory rate of 30 breaths per min. Physical exam findings were notable for altered mental status and mild intermittent bilateral wheezing. White blood cell (WBC) count was 12.4 × 10^9^/L and creatinine was elevated at 1.5 mg/dL from baseline of 0.7 mg/dL. Lactate was initially elevated at 6.2 mmol/L with a repeat lactate result of 1.2 mmol/L. Two sets of blood cultures were drawn on admission and the patient was started empirically on vancomycin, piperacillin–tazobactam 3.375 g every 8 h, and azithromycin 500 mg daily (Figure 1). Due to acute kidney injury (AKI) on presentation, vancomycin was dosed by level until Day 2. The patient was admitted for additional evaluation. Pertinent medical history includes history of polysubstance use disorder (intravenous fentanyl and cocaine), prior tricuspid valve endocarditis, untreated chronic hepatitis C, and housing instability.

On Day 2, both sets of blood cultures resulted as Gram-positive bacilli. The pathogen was not identified by the blood culture rapid polymerase chain reaction (PCR) testing panel. Empiric vancomycin, piperacillin–tazobactam, and azithromycin were continued. On Day 3, blood culture results identified *Bacillus cereus* in both sets. The infectious diseases (ID) specialist was consulted and piperacillin–tazobactam and azithromycin were discontinued. The patient was continued on vancomycin and doses were adjusted using area under the curve to minimum inhibitory concentration (AUC:MIC) ratio once the patient's AKI resolved on Day 2 (Table 1). Repeat blood cultures were drawn every other day to monitor for clearance of infection.

Transthoracic echocardiogram (TTE) and transesophageal echocardiogram (TEE) did not have any evidence of thrombus, vegetation, or abscess. Vertebral osteomyelitis and diskitis were ruled out with negative findings from magnetic resonance imaging (MRI) of the spine. An x-ray of the patient's right wrist did not demonstrate septic arthritis. Workup for metastatic sites of infection was negative.

*B. cereus* was detected in all subsequent blood cultures drawn on Days 3, 5, 7, 9, and 11 (Figure 1). Due to the persistently positive blood cultures, gentamicin was added to vancomycin for Gram-positive synergy on Day 11. Gentamicin was dosed at 80 mg every 8 h (3 mg/kg/day). Serum levels were monitored to target a trough less than 1 mcg/mL and a peak of 3–5 mcg/mL. On Day 12, the trough and peak resulted as 0.7 and 3.5 mcg/mL, respectively. Trough and peak levels on Day 15 were 0.5 and 4 mcg/mL, respectively. Creatinine remained stable throughout his course.

Final reports of blood cultures drawn on Days 13 and 15 confirmed clearance after the addition of gentamicin. Vancomycin and gentamicin were continued for 7 days from culture clearance until Day 19. Due to his persistent bacteremia and negative workup for metastatic sites of infection, the ID consulting provider recommended a total duration of 4 weeks of antibiotic therapy from the first day of culture clearance. To avoid discharging the patient with intravenous access, one dose of dalbavancin 1500 mg was administered on Day 20. Additionally, the patient was discharged on Day 20 with a 21-day course of oral levofloxacin 750 mg daily. As a result, the patient received dual coverage with dalbavancin and levofloxacin for 2 weeks after discharge and levofloxacin monotherapy for an additional week. An outpatient follow-up appointment with the ID provider was arranged. During the admission, the patient was initiated and maintained on buprenorphine–naloxone 8 mg twice daily, which was continued on discharge for his substance use disorder.

## 3. Discussion

This case describes a patient admitted with *B. cereus* bacteremia in the setting of IVDU. Due to its ability to form biofilms, severe *B. cereus* infections most commonly occur in patients who use intravenous drugs, have indwelling catheters, have traumatic or surgical wounds, or are immunocompromised [1]. While most isolates are susceptible to vancomycin, the patient in this case had persistently positive blood cultures despite vancomycin therapy.

There have been previous accounts of utilizing combination therapy with vancomycin and gentamicin for its potential synergistic effect against persistent *B. cereus* bacteremia. In one case, gentamicin was initiated after 11 days of vancomycin therapy [3]. In another two cases, gentamicin was initiated after six or seven days of vancomycin [9]. In two of these three cases, blood culture clearance occurred within 24 h [3, 9]. Time to clearance was not noted in the third case in which gentamicin was used [9]. These reports align with the progression of the current patient case in which the blood cultures cleared 2 days after gentamicin addition. In this case, the patient was started on gentamicin after 7 days of a therapeutic vancomycin dose based on AUC/MIC monitoring. In patients with persistent *B. cereus* bacteremia, vancomycin and gentamicin combination therapy may be considered as early as the sixth day of a therapeutic vancomycin regimen [9].

Therapeutic drug monitoring (TDM) was performed to confirm vancomycin dosing efficacy with a target AUC/MIC range of 400–600 mg∗hour/L [10]. Several previous studies have demonstrated efficacy of vancomycin for *B. cereus* bacteremia [3, 4, 11]. Only one study, however, has described TDM results when vancomycin therapy is used for *B. cereus* bacteremia [3]. Sasano and colleagues targeted a goal trough concentration of 10–20 mcg/mL [3]. In the current case, goal AUC/MIC was achieved by Day 4 and sustained throughout the vancomycin course. All AUC/MIC results correlated with troughs around or less than 10 mcg/mL. Notably, both cases required addition of gentamicin for blood culture clearance regardless of the method of vancomycin TDM used. Similar to Sasano and colleagues, gentamicin TDM was conducted using recommended peak and trough levels for its synergistic effect in infective endocarditis (IE) therapy as there is no guidance on TDM for Gram-positive bacilli [3, 12]. TDM in this case ensured safe and effective vancomycin and gentamicin use, including avoidance of nephrotoxicity.

In this case, the patient was transitioned to levofloxacin as an oral step-down option on discharge. Previous accounts of *B. cereus* bacteremia have utilized fluoroquinolones as combination therapy with vancomycin and as monotherapy on discharge. Wu and colleagues describe a patient with *B. cereus* bacteremia and infected aortic aneurysm who received 6 weeks of vancomycin followed by 4 weeks of oral fluoroquinolone monotherapy [13]. The oral fluoroquinolone agent administered was not specified. In another case of *B. cereus* bacteremia, the patient received 4 days of vancomycin and piperacillin/tazobactam, followed by a 14-day course of moxifloxacin and vancomycin [14]. The route of moxifloxacin administration was not specified. Overall, there are varying practices with use of fluoroquinolones for invasive *B. cereus* infections. In line with prior reports, the current patient received levofloxacin in combination with a glycopeptide. Based on existing literature, fluoroquinolones are typically given in combination therapy with vancomycin due to concerns for resistance. They may be given as monotherapy if isolate susceptibility is confirmed. Fluoroquinolones offer an oral option for *B. cereus* management, which is an important consideration for patients with a history of IVDU.

Given that injection drug use is often a barrier to outpatient parenteral antibiotic therapy, PWID with invasive infections frequently remain in the hospital for prolonged periods of time to complete IV antibiotic therapy for invasive infections [15]. There is emerging evidence that support alternative strategies for individual patient-centered decision making in challenging scenarios such as this [15]. The favorable pharmacokinetic and pharmacodynamic properties of novel long-acting medications such as dalbavancin may allow for treatment of disseminated infections in an outpatient setting, thus leading to shorter length of hospital stay, reduction in cost and healthcare resource use, and improved patient satisfaction. Dalbavancin has shown remarkable efficacy and good tolerability in different challenging scenarios such as IE, complicated bloodstream infections (BSI), and osteoarticular infections as described in the following [16].

Dalbavancin is a semisynthetic lipoglycopeptide antibiotic that is administered intravenously [17]. It has the same mechanism of action as vancomycin, binding to growing peptidoglycan chains to inhibit bacterial cell wall synthesis [18]. This allows for rapid and potent bactericidal activity against resistant Gram-positive bacteria [17]. Its long terminal half-life of approximately 14.4 days allows for weekly or biweekly administration, making it an attractive option for completion of therapy in PWID or those with barriers to IV access to avoid prolonged hospital admissions to receive intravenous antimicrobials for invasive infections [15]. The only approved indication for dalbavancin is the treatment of acute bacterial skin and skin structure infections (ABSSSIs) [16]. However, dalbavancin has gained recent attention for the off-label treatment of Gram-positive bacterial infections in conditions such as IE, vascular graft infections (VGIs), bone and joint infections (BJIs), prosthetic joint infections (PJIs), and BSIs [19].

Most of the published evidence supporting dalbavancin use for these indications has been retrospective and observational in nature. One multicenter, observational, retrospective study evaluated hospitalized patients with IE or BSI caused by GPC who received at least one dose of dalbavancin [20]. The most commonly isolated microorganisms were *Staphylococcus aureus* in BSI (49%) and coagulase-negative *Staphylococci* in IE (44.1%). The percentage effectiveness of dalbavancin to treat IE was 96.7%, and the clinical cure rate for BSI was 100%, with no recurrences or deaths during the follow-up [20]. The savings in hospital duration of stay was 636 days for BSI and 557 days for IE. Investigators concluded that dalbavancin is an effective consolidation therapy in clinically stabilized patients with IE and/or BSI, and that the use of dalbavancin was cost-effective due to reduced hospital stays [20]. Venturini and colleagues conducted a small pilot feasibility study assessing the efficacy and safety of a “single step” treatment strategy which included dalbavancin administration (1500 mg as a single dose), catheter removal, and early discharge in adult patients admitted to medical wards with catheter-related BSI (CRBSI) [21]. They enrolled 16 patients with CRBSI, most frequently caused by *Staphylococci* (25% methicillin-resistant strains). Ten out of 16 patients had been treated empirically before dalbavancin administration. None of the patients had adverse drug-related reactions, and at 30- and 90-day follow-up, no patients had been readmitted to the hospital due to bacteremia recurrence [21].

Given that much of the evidence in support of dalbavancin for disseminated infections is limited to retrospective studies or narrative reviews, Leanza and colleagues conducted a systematic review of dalbavancin efficacy as a sequential therapy for IE [19]. Their systematic review included 9 studies, all of which were observational. Native valve endocarditis was the most common type of IE found in the studies (128,263, 48.7%), followed by prosthetic valve endocarditis. Coagulase-negative *Staphylococci* were the most common pathogens isolated (30.1%), followed by *S. aureus, Enterococci* spp., and *Streptococci* spp. [19]. Five out of the nine studies documented a clinical failure rate of less than 10% and a favorable safety profile, demonstrating that dalbavancin appears to be a promising option for the consolidation therapy of IE [19].

Though there are limited prospective data in support of dalbavancin of completion of extended antibiotic courses in disseminated infections, one prospective, randomized, open-label, comparator-controlled trial evaluated the safety and efficacy of dalbavancin as a 2-dose regimen for osteomyelitis [22]. Eighty patients were randomized 7:1 to dalbavancin (1500-mg IV on Days 1 and 8) or standard of care (SOC) for osteomyelitis (oral or IV) per investigator judgment for four to six weeks. The primary endpoint was clinical response at Day 42, defined as recovery without need for additional antibiotics. *Staphylococcus aureus* was the most common pathogen (60% of patients). Clinical cure at Day 42 was seen in 65/67 (97%) and 7/8 (88%) of the patients in the dalbavancin group and SOC group, respectively. No patients discontinued the study drug due to treatment-emergent adverse effects [22]. The investigators concluded that the high microbiologic potency of dalbavancin combined with the prolonged tissue exposure at the site of infection and high clinical cure rates observed in this study make dalbavancin a convenient and effective option for an infection that has historically been burdensome for patients, providers, and the healthcare system [22]. In the current case, dalbavancin was utilized in conjunction with an oral fluoroquinolone to facilitate discharge in a patient with a history of IVDU, suggesting a potential role in therapy for Gram-positive infections. Due to limitations in available literature, further studies are needed to fully understand the use of dalbavancin in this context.

## 4. Conclusion

In a patient with a history of IVDU, the combination of vancomycin and gentamicin was effective in managing persistent *B. cereus* bacteremia. Due to challenges with maintaining prolonged intravenous access, the patient received a single dose of dalbavancin in addition to oral levofloxacin therapy to complete a 4-week antibiotic course following culture clearance. This case highlights the potential role of dalbavancin as an alternative treatment option in patients with challenges to maintaining prolonged intravenous access.

## Figures and Tables

**Table 1:** Therapeutic drug monitoring (TDM) of patient's vancomycin using area under the curve to minimum inhibitory concentration (AUC:MIC).

Day(s)	Serum creatinine (mg/dL)	Vancomycin dose, grams (g)	Level result (type of level, # hours drawn postdose)	AUC:MIC (mg∗h/L; goal 400–600)	Dose changes (if applicable)
1	1.5	1.5 g			Dose by level

2	0.9		8.8 (random, 10 h)		Start regimen with AUC:MIC TDM
		1.25 g Q12H			

3	0.8	1.25 g Q12H	8.7 (trough, 9 h)	384	Dose increased
		1.5 g Q12H			

4	0.7	1.5 g Q12H	8.5 (trough, 10.5 h)	448	No change

5–7	0.7	1.5 g Q12H			

8	0.7	1.5 g Q12H	9.5 (trough, 11.5 h)	499	No change

9	0.7	1.5 g Q12H			

10	0.6	1.5 g Q12H			

11-12	0.7	1.5 g Q12H			

13	0.7	1.5 g Q12H	9.6 (trough, 10 h)	478	No change

14–18	0.7	1.5 g Q12H			

19		1.5 g Q12H			Discontinued

20	0.7				Day of discharge

## Data Availability

Data sharing is not applicable to this article as no datasets were generated or analyzed during the current study.
